# Short-Term Feeding of Fibre-Enriched Biscuits: Protective Effect against Hepatotoxicity in Diabetic Rats

**DOI:** 10.1155/2015/868937

**Published:** 2015-12-02

**Authors:** Ochuko L. Erukainure, Osaretin A. T. Ebuehi, Folasade O. Adeboyejo, Olufunmilola O. Oladunmoye, Muhammad Aliyu, Okukwe C. Obode, Tosin Olasehinde, Gloria N. Elemo

**Affiliations:** ^1^Department of Food Technology, Federal Institute of Industrial Research, Oshodi 100261, Lagos, Nigeria; ^2^Department of Biochemistry, University of Lagos, Lagos, Nigeria; ^3^Product Development Programme, Nigeria Institute of Horticultural Research, Ibadan, Nigeria; ^4^Department of Biochemistry, Ahmadu Bello University, Zaria, Nigeria

## Abstract

The effects of fibre-enriched biscuit on biomarkers associated with hepatotoxicity in diabetic rats were investigated. Diabetes was induced by single intraperitoneal injection of alloxan monohydrate. Treatment lasted for 14 days after which the rats were sacrificed by cervical dislocation. Blood serum was analyzed to determine hepatic function enzymes. The liver was also analyzed to determine hepatic lipid profile and antioxidant enzymes. Induction of diabetes led to elevated levels of ALP, AST, and ALT. These were, however, significantly (*p* < 0.05) reduced in the fibre-enriched biscuit fed (treated) group. There was no significant difference in the serum bilirubin and total protein levels of the studied groups. Reduced albumin level was observed in the diabetic group; this was further lowered on feeding with fibre-enriched biscuits. Induction of diabetes led to increased hepatic level of cholesterol, triglyceride (TG), low density lipoprotein (LDL), and lipid peroxidation and decreased activities of glutathione (GSH), catalase (CAT), and superoxide dismutase (SOD) and HDL level. These were significantly (*p* < 0.05) reversed on feeding with fibre-enriched biscuit. This study portrays the protective effect of fibre-enriched biscuit on increased oxidative stress and hyperlipidemia in hepatic tissues of alloxan-induced diabetic rats.

## 1. Introduction

Diabetes is a chronic disease associated with high morbidity and mortality due to its complications and consequences [1]. It is characterized by chronic hyperglycemia and alterations of cellular homeostasis, which leads to diffuse vascular damage. The progression of this disease causes *β*-pancreatic cell dysfunction implicated in the development of cardiovascular diseases, neuropathy, nephropathy, and retinopathy [2]. Series of experimental investigations have shown that hyperglycemia-induced-oxidative stress via free radical generation and oxidative damage to cells plays a major role in these diabetic complications [1, 3]. Previous scientific investigations have shown associations between concentrations of hepatic function enzymes such as aspartate amino transferase (AST), alanine amino transferase (ALT), and the incidence of diabetes [4–6]. Wannamethee et al. [7] also suggested that elevated levels of hepatic enzymes could cause insulin resistance and other features of diabetic syndrome. Bakhshaeshi et al. [8] and Dey and Lakshmanan [9] have linked hyperglycemia-induced generation of reactive oxygen species (ROS) with development of diabetic liver injury and thus increased attention on antioxidants in the treatment and management of diabetes.

Food fibre has been a major component of human nutrition from time immemorial. Its significant role in the human physiology is well documented [10]. These varying physiological effects have been linked to the fact that dietary fibre is made up of different components, each with its own distinctive characteristics [10].

The beneficiary role of fibre inclusion in diets on the management of diabetes has been reported in several studies. Snacks enriched with fruit fibres were shown to lower blood glucose level and improve insulin sensitivity in diabetes [11]. Erukainure et al. [12, 13] also reported the protective effect of fibre-enriched snacks on oxidative stress in brain and renal tissues of diabetic rats. A reduction in serum level of hepatic function enzymes has been reported in diabetic rats fed on fibre-enriched biscuits [13], insinuating a possible curative role of food fibres against diabetic hepatotoxicity.

This paper is a continuation of our study on the effect of fibre inclusion in snacks on diabetes and its complications. This study investigates the effect of fibre-enriched biscuits on oxidative stress biomarkers and lipid profile in hepatic tissues as well as serum hepatic function enzymes.

## 2. Materials and Methods

### 2.1. Plant Materials

Banana (*Musa* species), oranges (*Citrus sinensis*), watermelon (*Citrullus lanatus*), pineapple (*Ananas comosus*), and pawpaw (*Carica papaya*) were purchased from Ketu fruit market, Ketu, Lagos, Nigeria.

### 2.2. Production of Fibre-Enriched Biscuits

After rinsing and peeling, juice was extracted from the oranges, pineapple, and watermelon, leaving behind the fibres. 400 g of each fibre was weighed and blended together with 400 g of pawpaw and banana, respectively, in a warring blender for 10 minutes to produce fibre paste [11].

Fibre-enriched biscuits were produced as described by Erukainure et al. [11].

### 2.3. Mineral Analysis

The biscuits were blended, from which 2 g was digested with concentrated nitric acid. The resulting solution was evaporated to dryness and dissolved in 100 mL deionized water. The solution was analyzed for mineral elements (calcium, magnesium, zinc, iron, sodium, potassium, and phosphorus) using an atomic absorption spectrophotometer (AAnalyst 200, PerkinElmer) and a flame photometer (Jenway PFP7) [14].

### 2.4. Phytochemical Analysis

Phytochemical properties (alkaloid, flavonoids, phenol, anthocyanin, saponins, and carotenoids) of the developed biscuits were determined using standard methods [15].

### 2.5. Animals

Eighteen male albino rats of Wister strain weighing about 150–200 g were used for the study. They were fed on standard rat pellet diet (Ladoke feeds) and allowed to adapt for one week. They were provided water* ad libitum* and maintained under standard laboratory conditions of natural photoperiod of 12 hr light-dark cycle. The animals used in the present study were maintained in accordance with the approval of the Animal Ethical Committee, University of Lagos, Lagos, Nigeria. The approval number from the Animal Institutional Ethical Committee is UL/CMUL/IEC 2011/1003.

### 2.6. Induction of Diabetes and Experimental Design

Diabetes was induced by a single intraperitoneal injection of 180 mg/kg* of alloxan* monohydrate in normal saline water in a volume of about 3 mL. After 72 hours of alloxan injection, the diabetic rats (glucose level > 150 mg/dL) were separated and used for the study.

The rats were divided into three groups, each consisting of six animals:

 Group 1: normal rats + pelletized mouse chows.
 Group 2: diabetic (untreated).
 Group 3: diabetic + fibre-enriched biscuits.

The rats were monitored daily for food and water intake and body weight. Blood glucose levels of the rats were monitored on weekly basis with a glucometer. Treatment lasted for 14 days. At the end of the feeding trials, the rats were fasted overnight and sacrificed by cervical dislocation.

### 2.7. Collection of Blood and Preparation of Serum

Blood was collected from each rat by cardiac puncture and transferred into clean plain centrifuge tube bottles. Part of the blood sample was centrifuged at 3000 rpm for 10 minutes, and the serum (supernatant) was transferred into labeled sample bottles. They were stored at 4°C to maintain enzyme activity.

### 2.8. Determination of Hepatic Function Enzymes

Blood serum was used for the evaluation of hepatic function biomarkers which covers for alkaline phosphatase (ALP), aspartate aminotransferase, (AST), alanine aminotransferase (ALT), total bilirubin (T BIL), and total protein (TP DIL) using commercial kits from Randox Laboratories, UK, according to the manufacturer's protocol.

### 2.9. Preparation of Tissue Homogenates

Hepatic organs were harvested, rinsed in ice-cold 1.15% KCl solution to wash off excess blood, blotted dry with filter paper, and weighed. They were homogenized in phosphate buffer (0.01 M) and centrifuged at 10,000 rpm for 15 min in an ultracentrifuge at a temperature of −2°C. The supernatant was decanted and stored at −4°C for subsequent analysis. Each time the supernatant was outside the freezer, it was kept in ice bags.

### 2.10. Determination of Oxidative Stress Parameters in Tissue Homogenates

Lipid peroxidation was determined by measuring malondialdehyde (MDA) formed by thiobarbituric acid reaction (TBAR) [16]. Catalase (CAT) activity was estimated by measuring the rate of decomposition of H_2_O_2_ [17]. The level of superoxide dismutase (SOD) activity was determined by the method of Misra and Fridovich [18], while the method of Ellman [19] was adopted in estimating the activity of reduced glutathione (GSH).

### 2.11. Determination of Lipid Parameters in Tissue Homogenates

Tissue total cholesterol (TC), triglyceride (TG), and high density lipoprotein (HDL) were measured by enzymatic colorimetric method using Randox kits according to manufacturer's protocol. The concentration of low density lipoprotein (LDL) cholesterol was calculated by the formula of Friedwald et al. [20].

### 2.12. Statistical Analysis

To address the biological variability, each set of experiments was repeated at least three times (*n* = 3) for phytochemical analysis and six times for experimental rats (*n* = 6). Differences between the groups were analyzed by one-way analysis of variance (ANOVA) with the aid of SPSS software (SPSS Inc., Chicago, IL, USA) standard version 17. The *p* values of <0.05 were considered statistically significant for differences in mean using the least of significance difference, and data were reported as mean ± standard deviation.

## 3. Results

Table 1 depicts the mineral composition of the developed fibre-enriched biscuits. A very high concentration of iron was observed; this was followed by calcium and magnesium, respectively. Lower concentrations of zinc, phosphorus, and sodium were also observed, with sodium being the lowest.

Phenol had the highest concentration; this was followed by carotenoids and flavonoids as shown in Table 2. The concentration of alkaloid was rather very low as compared to other studied phytochemicals.

Induction of diabetes led to elevated levels of ALP, AST, and ALT as depicted in Table 3. This was significantly (*p* < 0.05) reduced on feeding with the formulated biscuit. No significant difference was observed in the total bilirubin and protein levels in the studied groups.

**Figure 1:**
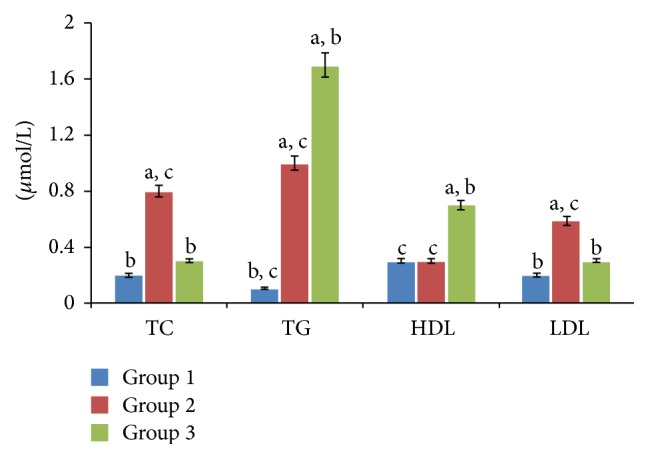
Lipid profile of hepatic tissues of experimental groups. Note: values = mean ± SD; *n* = 6. (a) Statistically significant (*p* < 0.05) as compared with group 1; (b) statistically significant (*p* < 0.05) as compared with group 2; (c) statistically significant (*p* < 0.05) as compared with group 3.

The lipid profile of the experimental groups is shown in Figure 1. A significantly (*p* < 0.05) increased level of total cholesterol, triglyceride, and LDL and decreased HDL levels were observed on induction of diabetes. These were significantly (*p* < 0.05) reversed on feeding with the formulated biscuit.

A significant (*p* < 0.05) decrease in the activities of glutathione (GSH), superoxide dismutase (SOD), and catalase in the hepatic tissue was also observed on induction of diabetes (Table 4). However, feeding on the formulated biscuit led to a significant (*p* < 0.05) increase in their activities.

**Figure 2:**
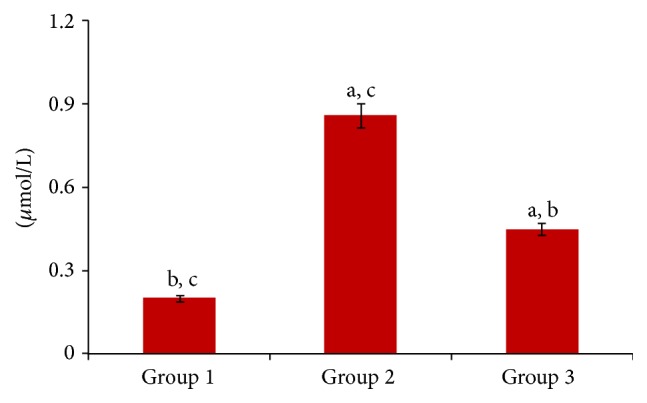
Lipid peroxidation in hepatic tissues of experimental animals. Note: values = mean ± SD; *n* = 6. (a) Statistically significant (*p* < 0.05) as compared with group 1; (b) statistically significant (*p* < 0.05) as compared with group 2; (c) statistically significant (*p* < 0.05) as compared with group 3.

Similarly, induction of diabetes led to a significant (*p* < 0.05) increase of MDA production as shown in Figure 2. However, feeding with the biscuit significantly (*p* < 0.05) reduced its level.

## 4. Discussion

This present study was designed to assess the protective potential of fibre-enriched biscuit against diabetic induced hepatotoxicity. Hepatic tissues have been implicated as a risk factor in the pathogenesis of diabetes due to inflammation via oxidative damage and insulin resistance (type 2 diabetes) as a result of hepatic dysfunction [4, 21]. Inflammation of the liver allows hepatic enzymes to leak out of the cells into the blood stream. The observed increase in ALP, AST, and ALT levels in the serum of the diabetic rats shows an occurrence of hepatic injury (Table 2). The reduced levels of these enzymes on feeding with the formulated biscuit may indicate stabilization of hepatic plasma membrane and repair of hepatic tissues. This effect could be attributed to the presence of the studied minerals and phytochemicals in the biscuit.

It has been established that high levels of TC, TG, and LDL are major risk factors associated with diabetic complications [22]. Of such complications is hepatic steatosis [23]. It is characterized by an increased rate of hepatic triglyceride synthesis and VLDL particle production, which results in secondary abnormalities of low HDL and increased LDL particle number and density [24]. In this study, the observed reduction of elevated levels of TC, TG, and LDL as well as increased HDL on feeding with the formulated biscuit is of great significance and indicates an antilipidemic activity. This antilipidemic activity can be attributed to the fibre enrichment. Food fibres have been reported to reduce hyperlipidemia by binding and reducing the production of bile acids [25]. They have also been suggested to increase the generation of propionate, which decrease cholesterol levels and stall cholesterol synthesis [26]. Saponins and anthocyanins have also been reported to reduce LDL, TG, and TC levels in dyslipidemic subjects [27].

Oxidative stress has also been linked with the development and progression of diabetes via the deleterious effects of free radicals. Effectiveness of antioxidant enzymes can help to prevent oxidative damage induced by free radicals. The concentrations of these enzymes increase in response to free radical attack. Glutathione (GSH) is majorly produced in all cells and has the capacity to reduce oxidative stress by minimizing lipid peroxidation and preventing hydrogen-peroxide induced cell death [28]. Similarly SOD quenches the action of superoxide thereby preventing its conversion to hydrogen peroxide which is capable of initiating lipid peroxidation [29]. Catalase initiates the destruction of hydrogen peroxide in the cells thereby neutralizing its effects [30]. It is located in peroxisomes and found essentially in all aerobic cells [31]. The decreased activities of GSH, SOD, and CAT on induction of diabetes were significantly (*p* < 0.05) reversed on feeding with the formulated biscuit. This indicates an antioxidant activity. The increased lipid peroxidation level on induction of diabetes indicates an occurrence of oxidative stress. The presence of fibres may contribute to the observed antioxidant activity of the formulated biscuit by reducing blood glucose and increasing serum insulin levels as reported by Erukainure et al. [12], thereby reducing the amount of blood glucose available for oxidation. The reduced level on feeding with the formulated biscuit further corroborates its antioxidant potential. The antioxidant activity can also be attributed to the studied phytochemicals and minerals. The antioxidant properties of the studied phytochemicals especially phenols and flavonoids are well known [32]. Zinc is an important component of SOD and its deficiency greatly affects the activity of this enzyme [33]. Zinc also stabilizes cell membranes possibly by displacing bound transition metal ions, thus preventing peroxidation of membrane lipids [34]. Although iron overload has been implicated in progression of oxidative stress via the Fenton reaction, it is however a major cofactor for catalase [35].

Based on the results, this study can be summarized as follows: induction of diabetes led to an onset of oxidative stress in hepatic tissues leading to peroxidation of the membrane lipids and alteration of hepatic lipid profile. This in turn led to cellular damage and membrane rupture, causing a release of AST, T BIL, ALT, ALB, ALP, and TP DIL from the cytoplasm into the blood stream. The formulated biscuit may therefore protect against diabetic induced hepatotoxicity by (1) increasing the activities of GSH, SOD, and catalase, (2) attenuating lipid peroxidation of hepatic plasma membrane lipid, and (3) reducing total cholesterol level and subsequently increasing HDL level in hepatic tissues.

## 5. Conclusion

This study portrays the protective effect of fibre-enriched biscuit on increased oxidative stress and hyperlipidemia in hepatic tissues of alloxan-induced diabetic rats as revealed by the increased antioxidant biomarkers and reduced hepatic function enzymes levels, thus, indicating its potential in the management and treatment of hepatotoxicity in diabetes.

## Figures and Tables

**Table 1:** Mineral composition of developed fibre-enriched biscuit.

Minerals	Concentration (mg/100 g)
Sodium	10.58 ± 0.23
Zinc	30.05 ± 0.38
Phosphorus	55.05 ± 0.30
Potassium	100.58 ± 0.19
Magnesium	210.53 ± 0.24
Calcium	321.5 ± 0.24
Iron	509.36 ± 0.22

Values = mean ± SD; *n* = 3.

**Table 2:** Phytochemical composition of developed fibre-enriched biscuit.

Phytochemicals	Concentration (mg/mL)
Alkaloid	5.62 ± 0.36
Saponin	17.69 ± 0.35
Anthocyanin	24.08 ± 0.24
Flavonoids	40.13 ± 0.44
Carotenoids	40.15 ± 0.33
Phenol	65.29 ± 0.31

Values = mean ± SD; *n* = 3.

**Table 3:** Hepatic function enzymes of experimental groups.

Parameters	Group 1	Group 2	Group 3
AST (*µ*/L)	83.56 ± 17.91^b,c^	107.72 ± 10.21^a,c^	56.90 ± 3.05^a,b^
T BIL mg/dL	6.50 ± 1.34	6.22 ± 1.19	6.03 ± 0.22
ALT (*µ*/L)	21.80 ± 3.46	29.26 ± 1.82^c^	19.18 ± 3.79^b^
ALB (g/dL)	14.86 ± 0.83^c^	11.15 ± 1.65	8.48 ± 2.58^a^
ALP (*µ*/L)	96.34 ± 20.11^b^	203.34 ± 53.48^a,c^	110.40 ± 9.76^b^
TP DIL (g/dL)	36.76 ± 2.05	36.94 ± 2.25	31.72 ± 5.38

Values = mean ± SD; *n* = 6. ^a^Statistically significant (*p* < 0.05) as compared with group 1; ^b^statistically significant (*p* < 0.05) as compared with group 2; ^c^statistically significant (*p* < 0.05) as compared with group 3.

**Table 4:** Antioxidant activities of hepatic tissues of experimental groups.

Parameters	Group 1	Group 2	Group 3
GSH (U/mg protein)	13.46 ± 0.71^b,c^	4.92 ± 1.02^a^	7.89 ± 1.66^a^
SOD (U/mg protein)	221.03 ± 5.14^b,c^	97.28 ± 3.85^a,c^	164.00 ± 9.48^a,b^
Catalase (U/mg protein)	1478.52 ± 34.45^b^	651.27 ± 26.38^a,c^	1097.05 ± 63.45^b^

Values = mean ± SD; *n* = 6. ^a^Statistically significant (*p* < 0.05) as compared with group 1; ^b^statistically significant (*p* < 0.05) as compared with group 2; ^c^statistically significant (*p* < 0.05) as compared with group 3.

## Abbreviations

CAT: Catalase
GSH: Reduced glutathione
HDL: High density lipoprotein
LDL: Low density lipoprotein
MDA: Malondialdehyde
SOD: Superoxide dismutase
TBAR: Thiobarbituric acid reaction
TC: Total cholesterol
TG: Triglyceride.
